# A Comprehensive Review of Trichosporon spp.: An Invasive and Emerging Fungus

**DOI:** 10.7759/cureus.17345

**Published:** 2021-08-21

**Authors:** Vibha Mehta, Charu Nayyar, Neelam Gulati, Nidhi Singla, Sunvir Rai, Jagdish Chandar

**Affiliations:** 1 Infectious Diseases, Institute of Liver and Biliary Sciences, New Delhi, IND; 2 Clinical Microbiology, Medanta SN Superspeciality Hospital, Sriganganagar, IND; 3 Clinical Microbiology, Government Medical College & Hospital, Chandigarh, Chandigarh, IND; 4 Microbiology, Government Medical College & Hospital, Chandigarh, Chandigarh, IND; 5 Preventive and Social Medicine, Government Medical College, Patiala, Patiala, IND

**Keywords:** trichosporon, antifungal susceptibility, invasive trichosporonosis, minimum inhibitory concentration, emerging yeast, trichosporon asahii

## Abstract

*Trichosporon *species are basidiomycetous yeast-like organisms found ubiquitous in nature. They are increasingly been recognized as opportunistic pathogens capable of causing life-threatening invasive diseases (trichosporonosis), especially in immuno-suppressed patients and rarely in immuno-competent patients too. Earlier multiple members of the genus *Trichosporon* were clubbed together as *T. beigelli* but after the advent of molecular techniques, more than 50 different subspecies and around 16 different strains causing human diseases are reported. It is known to cause a wide range of diseases, from superficial to probable and proven invasive diseases to summer hypersensitivity. The ability of *Trichosporon* strains to form biofilms on implanted devices, glucuronoxylomannan in their cell walls, and production of proteases and lipases lead to the virulence of this genus. This ubiquitous fungus exhibits intrinsic resistance to echinocandins, variable minimum inhibitory concentrations (MIC) for amphotericin B, and moderate susceptibility to fluconazole and Itraconazole, which are the commonly used anti-fungal agents for any invasive fungal infections which lead to the re-emergence of this notorious yet neglected pathogen and hence the reports of breakthrough infections among patients receiving these antifungals. This review is to understand the epidemiological, clinical details, and antifungal susceptibility pattern of various *Trichosporon* infections and it highlights the importance of early detection and treatment for this emerging yeast and also will add to the ongoing surveillance for the anti-fungal susceptibility pattern for this fungus.

## Introduction and background

The word Trichosporon, a combination of two Greek words Trichos (hair) and sporon (spores) was first discovered by Biegel in 1865, who observed that this organism caused benign hair infection. The superficial fungal infection caused by the genus *Trichosporon* started to be known as white piedra (meaning “stone”) because of the presence of hard and irregular nodules along the hair [1]. But over the last few decades, *Trichosporon spp.* has increasingly been recognized as opportunistic pathogens capable of causing life-threatening invasive diseases, not just in immunosuppressed but also in immunocompetent patients [1].

In 1902, all *Trichosporon* species were designated as *Trichosporon beigelii*, yeast containing arthrospores, by Vuillemin [1]. Another species was discovered from a patient with pruritic cutaneous lesions in 1909 by Buermann et al., which was designated by Ota in 1926 as *Trichosporon cutaneum* [2]. Further in 1942, two scientists Diddens and Lodder found these two species to be the same. Thus the use of two terminologies for the same organism i.e *T. beigelli* preferred by the physicians and *T. cutaneum* by the mycologists [2].

The above nomenclature was considered inappropriate and thus abandoned when in 1992 after Gueho et al. explained that the two species to be completely non-related as detected by the ubiquinone system and percentage of GC content. Eventually, in 1994, it was defined that the genus *Trichosporon* mainly included six major pathogenic species: *T. asahii, T. asteroides, T. cutaneum, T. mucoides, T. inkin* and *T. ovoides* [3]. Even more recently *T. loubieri*, and *T. pullulans* have been reported as major causes of invasive infection. It was concluded that the genus *Trichosporon* has 50 species, four clades, which includes 16 species of clinical relevance [1].

## Review

Pathogenicity

*Trichosporon* is a part of the normal flora of the human skin, vagina, and gastrointestinal tract [4]. The infections caused by *Trichosporon spp.* can be broadly categorized as:

a) Superficial Infection

The most common clinical presentation of superficial *Trichosporon* infection is benign irregular nodules on the hair shaft lesions called white piedra [4]. Despite the “stony” name, nodules have a soft texture and are loosely attached to the shaft, and vary from white to light brown in color. This disease has a primary predisposition towards children and young adults from tropical and temperate areas and mostly affects females using headbands frequently [5]. White piedra is mainly caused by *T. cutaneum, T. inkin, T. ovoides, and T. loubieri *[3]. It may affect the scalp, beard, mustache, eyebrows, axilla, and even genital hairs [6]. Another common presentation of superficial infection caused by *Trichosporon* is onychomycosis, and the common causative agent is *T. cutaneum* [7].

b) Invasive Infections

In patients with underlying history of hematological cancer, *Trichosporon spp.* is second common to *Candida* in causing disseminated yeast infections. The mortality attributed to *Trichosporon spp.* in such patients, in spite of the antifungal therapy is very high and ranges from 50-80% [8]. Breakthrough trichosporonosis has been colossally reported commonly in immunocompromised patients especially after the administration of ineffective antifungal treatment by amphotericin B, echinocandins, and rarely triazoles [1]. Disseminated trichosporonosis may affect a wide array of organs and the presentation may vary from brain abscess, meningitis, endophthalmitis, pneumonia, soft tissue lesions, lymphadenopathy, endocarditis, arthritis, esophagitis, liver, and splenic abscess, or even uterine infections [1].

As per the European Organization for Research and Treatment of Cancer/Invasive Fungal Infection Cooperative Group (EORTC/IFICG) and the National Institute of Allergy and Infectious Disease Mycoses Study Group (NIAID/MSG), invasive trichosporonosis has been classified as follows [1,9]:

1. Proven invasive trichosporonosis - patients presenting with at least one of the following criteria:

 i) Blood cultures yielding *Trichosporon* species in patients with temporally related clinical signs and symptoms of infection

(ii) Cerebrospinal fluid (CSF) cultures yielding *Trichosporon* species,

(iii) Biopsy specimens that are culture positive and present histopathological evidence of fungal elements compatible with *Trichosporon* species.

2. Probable invasive trichosporonosis - patients presenting all of the following criteria:

 (i) Presence of at least one host factor (therapy with immunosuppressive drugs, neutropenia, or persisting fever despite therapy with appropriate broad-spectrum antibiotics

(ii) One microbiological criterion (culture or presence of fungal elements compatible with *Trichosporon* in a suspect sample)

(iii) One major clinical criterion (imaging or cytobiochemical findings) is consistent with infection.

*T. asahii* is the most common causative agent of invasive trichosporonosis [1]. Invasion occurs either through the exogenous route when the organism enters through the central lines and catheters through colonized skin or endogenously by translocation through the gut in immunocompromised patients [10]. In very low birth weight (VLBW) babies, the organism may enter either through the mother's genital region or through the hands of health care workers [11].

c) Summer type hypersensitivity

*Trichosporon* is also attributed as the major etiological agent of summer-type hypersensitivity (SHP) in Japan and *T. cutaneum* has been named as the main causative agent [12].

Virulence factors

a) Biofilm

*Trichosporon* adheres to the implanted devices and forms biofilms. This biofilm helps in the invasion of the surface by evading the host immune response as well as the effect of antifungal drugs. The kinetics of biofilm formation in *Trichosporon* was first studied by Di Bonaventura et al. on strains that were obtained from blood cultures of patients with hematological malignancies. With the help of electron microscopy, they demonstrated that the biofilm formation of *T. asahii *strains was similar to that produced by *Candida spp.* after a 30min incubation. After 72 hrs incubation, *T. asahii* showed morphologies such as budding yeast cells and filamentous forms, engulfed in an extracellular polysaccharide matrix which was the main constituent of the ultrastructure of a mature biofilm [13]. Various imaging sections taken across the thickness of biofilm revealed that the thickness to be somewhere between 25 to 40 µm. They simultaneously compared the antimicrobial susceptibility pattern of planktonic cells to biofilm cells of *Trichosporon spp.* for various antifungals like amphotericin B, caspofungin, fluconazole, and voriconazole and found that biofilm cells of *T.asahii* were highly resistant to all antifungals tested (MIC 1,024 g/ml) and were up to 16,000 times more resistant than planktonic cells to voriconazole (MIC =0.06 g/ml) [13].

b) Enzymes

Proteases and phospholipases act by disrupting the proteins of the host cell membrane and thus help in the invasion of the fungus. Their role in pathogenicity varies according to their level of expression and host immune response [14]. Chen et al. in 1994 isolated and purified two lipase enzymes (lipase I and II) from *T. fermentans* and exhibited that these enzymes were stable even after incubation at 30°C for 24 h over a pH range of 4.0 to 8.0 [15].

c) Cell Wall Components

Similar to *C. neoformans*, members of the genus *Trichosporon* also produce glucuronoxylomannan (GXM) in their cell walls. GXM is a 1,3-linked mannan backbone attached to short side chains of 1,4-linked mannose and 1,2-linked xylose residues by substitution of two or four portion of the 1,3-linked mannose residues of the main molecule [16]. It has been demonstrated that GXM enervated the phagocytic nature of neutrophils and monocytes against the fungus [17]. Its presence is associated with infection in *C. neoformans*, however, this correlation has not been yet established for *Trichosporon* [1].

d) Adapting Technique

Members of the genus *Trichosporon* are ubiquitous in nature and so have a wide range of habitats and environments. This led to an assumption that they are yet less specialized as human pathogens and therefore it has been suggested by various authors that this genus is still in the process of adaptation to the animal host. This hypothesis explains the prominent differences in genome size of *Trichosporon* and *Malassezia*. However, extensive research is needed to establish the *Trichosporon*-host interactions [18].

Laboratory diagnosis

a) Phenotypic Identification

Yeast-like colonies are isolated on sabouraud dextrose agar. The colonies are cream-colored, however, may darken to yellowish-grey. They are highly wrinkled; the center of the colony becomes heaped and appears folded. The colonies may adhere to and crack the agar [4]. On cornmeal-tween 80 agar, true or pseudohyphae with blastoconidia and arthroconidia singly or in short chains are demonstrated. All species of *Trichosporon* hydrolyze urea thus differentiating it from *Geotrichum*, which also produce arthroconidia [4]. 

The commercial systems most commonly used rapid yeast identification in clinical laboratories are both non-automated like API 20C AUX (bioMe´rieux, France), ID 32C (bioMe´rieux, France), and RapID Yeast Plus system (Innovative Diagnostic System, Norcross, GA) and automated like Vitek Systems (bioMe´rieux, Vitek, Hazelwood, MO) and Baxter Microscan (Baxter Microscan, West Sacramento) [1,19].

b) Molecular Methods

Though expensive for routine use in developing nations, DNA-based PCR methods are more precise and lead to the accurate identification of *Trichosporon* species. The targets for species identification and phylogenetic studies are ribosomal genes including alternating conserved regions (D1/D2 region of the 28S rDNA) and variable regions (ITS and IGS1 regions) [20,21]. Conserved D1/D2 region of the 28S rDNA) were amplified using six primer sets to make them more sensitive, specific and to shorten the identification span to five hours. Thus the wisest approach is to use these tools as a first screening tool for *Trichosporon* species in biological samples [22]. Other molecular markers such as mitochondrial cytochrome b (Cyt b) have also been used for the detection of *Trichosporon* species. Another region of 396 bp fragment of Cyt b gene was amplified by Biswas et al. in 23 fungal strains and observed that there were 141 variable nucleotide sites (35.6%) among various *Trichosporon* strains [23].

c) Antigen Detection

Beta D glucan and galactomannan are being increasingly used as biomarkers for the early diagnosis of certain invasive fungal infections such as candidiasis and aspergillosis [24]. However, its role in the diagnosis of invasive trichosporonosis is still uncertain. Research from Japan analyzed 33 cases of *Trichosporon Fungemia* and demonstrated that only 50% of patients had a single positive test at the time of admission and a few had a positive test before positive blood cultures were obtained [25]. It was demonstrated by Lyman et al. that all *Trichosporon* strains produced a detectable amount of GXM in their cell wall and so this GXM cross-reacts with *C. neoformans* antigen in sera of patients with invasive trichosporonosis. If structural and serological properties of *Trichosporon* GXM can be studied properly and differentiated from *C. neoformans*, it can act as a useful tool in early and accurate diagnosis of invasive *Trichosporon* infection [26].

d) Proteonomics

Proteonomics works on the principle of protein content detection of a sample using mass spectrometry (MS) [1]. Many software are available for this study, amongst which MALDI BioTyper 2.0 is the most commonly used. This software possesses approximately 3700 profiles in its database. Among these, 274 (7.40%) represent fungus, of these 11 (4.01%) are related to *Trichosporon* species. Out of which six are *T. asahii, T. mucoides, T. debeurmannianum, T. inkin, T. ovoides,* and *T. cutaneum* and two are *Trichosporon* species. The biggest advantage of this method is that it is the least time-consuming. An isolate can be processed and identified within 2 min, making this method a next-generation tool in organism identification [27]. MALDI-TOF MS promises to be a great tool in organism identification by producing protein fingerprints [1].

The accuracy of detection of *Trichosporon* species by MALDI-TOF MS was tested by Bader et al. using two commercially available software, MALDI BioTyper2 (Bruker Daltonics) and Saramis (AnagnosTec), for 1,192 clinical yeast isolates which were previously identified by morphological characterization and biochemical tests (API 20 C AUX and ID 32 C galleries) [28]. The consilience among these three different identification methods was 95.1%, and all isolates of *T. asahii* were identified correctly by these three techniques [28].

e) Genotyping of Trichosporon Species

Using the IGS1 sequencing, nine different genotypes of *T.asahii*, the most important clinical species, have been described [29]. Amongst all the genotypes, genotype 1 is the most predominant type all over the world, ranging from 45 to 80%, except for the US where it has never been isolated. Genotypes 3 and 5 are abundant in the United States [1].

Antifungal susceptibility testing & antifungal therapy

Antifungal susceptibility testing of the genus *Trichosporon* for evaluating the effect of various antifungal drugs is being done as per the Clinical and Laboratory Standards Institute (CLSI) broth microdilution method (2008), which is currently been standardized for *Candida* species and *C. neoformans*. European Committee for Antimicrobial Susceptibility Testing (EUCAST) broth microdilution method, a recommendation originally proposed for the genus *Candida* is also being used though no clear-cut breakpoints have been identified by them [30,31].

Some authors have questioned the accuracy of the broth microdilution method for detecting isolates with resistance to amphotericin B quoting the narrow MIC variation seen in them. Hence clear-cut MIC breakpoints have not been confirmed for amphotericin B [32]. Hence, sensitivities of various species of the genus *Trichosporon* to commonly used antifungal drugs are still not known. Various studies have found that *T. asahii* strains are more sensitive to higher azoles and comparatively resistant to amphotericin B than other yeast-like species [1].

All tested *T. asahii* strains have demonstrated a very high MIC of ≥2μg/ml for amphotericin B. Many recent studies have shown a limited role of amphotericin B in vitro and in vivo. Almost all isolates showed resistance to *Echinocandins* in various studies. However, strains of *Trichosporon* have shown good susceptibility for voriconazole and posaconazole [1,33]. Topical azoles have shown promising results in superficial infections, along with proper personal hygiene which prevents recurrence [31]. For systemic infections, triazoles have better efficacy than amphotericin B. Voriconazole is considered the drug of choice in neutropenic patients with disseminated trichosporonosis [12] [33]. Antifungal treatment in such patients should be continued till the resolution of neutropenia, fever, radiological findings, and negative culture report. *Echinocandin* is not recommended in the treatment of *Trichosporon* as its role is very limited. 5flucytosine (5 FC) also does not have any activity against *Trichosporon*. However, some reports suggest that combination therapy of 5FC and amphotericin B show good results in the treatment of trichosporonosis [34]. Authors also suggest that a combination of echinocandin along with amphotericin B or azoles have shown synergistic antifungal effects [35], but still clear-cut guidelines are awaited, and review studies like these help in deciding the cut off values. The biochemical profile and growth characteristics for *Trichosporon* identification (Table 1) [36].

**Table 1:** Biochemical profile and growth characteristics of various Trichosporon spp. [36]

Strains	T. asahii	T. inkin	T. mucoides	T. ovoides	T. asteroides	T. cutaneum
Tests						
Sugar assimilation						
Myo- inositol	V	+	+	+	+	+
L- arabinose	+	-	+	-	+	+
Sorbitol	V	V	+	V	V	V
Melibiose	-	-	+	-	+	-
Growth at 37°C	+	+	+	V	V	-
Growth in the presence of 0.1% cyclohexamide	N	V	N	N	N	N

and MICs of *Trichosporon spp.* for different antifungals studied by various authors have been tabulated in Table 2 for reference [37-47].

**Table 2:** MIC's of Trichosporon isolates in various studies from 2010-2021 MIC: Minimum Inhibitory Concentrations, CLSI: Clinical and Laboratory Standards Institute, EUCAST: European Committee for Antimicrobial Susceptibility Testing

References		Kalkanci et al. [37]	Leme’s et al. [38]	Sun Wei et al. [39]	Yang et al. [40]	Arabatzis et al. [41]	Taverna et al. [42]	Montoya et al. [43]	Almeida et al. [44]	Rastogi et al. [45]	Singh S et al. [46]	Mehta V et al. [47]
Year		2010	2010	2012	2013	2014	2014	2015	2016	2016	2019	2021
Country		Turkey	Brasil	Beijing	Taiwan	Greece	Argentina	Mexico	Brasil	India	India	India
No of isolates		107	34	12	32	42	41	39	9	31	24	46
Method Used		ASTY Colorimetric method	CLSI	CLSI	CLSI	CLSI EUCAST	EUCAST	CLSI	CLSI	CLSI	CLSI	CLSI
Incubation period		-	24 hours and 48 hours	24-48 hours	48hours	CLSI=48 hours EUCAST=24 hours	24 -48 hours	24 hours	-	-	24 hours	-
Drugs												
Amphotericin-B (µg/ml)	MIC Range	0.125-4	24 hours: 0.06-64 48 hours: 0.06-64	0.25-1	0.5-4	CLSI: 0.032-64 EUCAST: 0.064-32	0.25-4	0.5-16	0.06-1	0.25->- 64	0.125–16	0.25-≥16
	MIC 50	1	24 hours: 2 48 hours: 4	-	0.5	CLSI: 2 EUCAST: 2	-	2	-	16	-	16
	MIC 90	2	24 hours: 4 48 hours: 64	-	2	CLSI: 32 EUCAST: 16	-	4	-	>16	-	16
5-Flucytosine (µg/ml)	MIC Range	0.125-32	24 hours: 0.25-32 48 hours: 0.25-64	1-Aug	-	-	-	Apr-64	-	-	-	0.125-≥64
	MIC 50	16	24 hours: 2 48 hours: 4	-	-	-	-	16	-	-	-	2
	MIC 90	16	24 hours: 4 48 hours: 16	-	-	-	-	32	-	-	-	8
Fluconazole (µg/ml)	MIC Range	Apr-64	24 hours: 0.5-8 48 hours: 0.05-16	0.5-4	Feb-16	CLSI: 1-64 EUCAST: 0.5-64	Jan-64	0.125-16	0.25-4	2->64	0.06–256	≤0.125-32
	MIC 50	8	24 hours: 2 48 hours: 4	-	2	CLSI: 8 EUCAST: 8	-	0.5	-	2		4
	MIC 90	16	24 hours: 8 48 hours: 8	-	4	CLSI: 64 EUCAST: 64	-	1	-	16		8
Itraconazole (µg/ml)	MIC Range	0.25-2	24 hours: 0.5-32 48 hours: 0.5-64	0.03-0.25	-	CLSI: 0.25-32 EUCAST: 0.25-32	0.03-0.5	-	0.03-0.06	0.25-16	-	0.125-0.25
	MIC 50	1	24 hours: 2 48 hours: 4	-	-	CLSI: 2 EUCAST: 1	-	-	-	0.25	-	0.0313
	MIC 90	2	24 hours: 8 48 hours: 16	-	-	CLSI: 32 EUCAST: 32	-	-	-	0.5	-	0.125
Voriconazole (µg/ml)	MIC Range	0.03-0.25	24 hours: 0.03-4 48 hours: 0.03-4	0.03-0.12	0.0313-8	CLSI: 0.64-32 EUCAST: 0.064-32	0.03-0.5	0.03-1	-	0.12-4	0.0616–4	≤0.0313-0.5
	MIC 50	0.125	24 hours: 0.5 48 hours: 1	-	0.0313	CLSI: 1 EUCAST: 1	-	0.03	-	0.12	-	0.0313
	MIC 90	0.25	24 hours: 1 48 hours: 2	-	0.0625	CLSI: 32 EUCAST: 32	-	0.03	-	4	-	0.25
Posaconazole (µg/ml))	MIC Range	-	-	-	-	CLSI: 0.032-16 EUCAST: 0.064-32	0.015-1	0.03-0.5	-	0.25-4	0.0616–32	≤0.0313-2
	MIC 50	-	-	-	-	CLSI: 1 EUCAST: 1	-	0.06	-	0.5	-	0.125
	MIC 90	-	-	-	-	CLSI: 4 EUCAST: 4	-	0.25	-	2	-	0.5
Caspofungin (µg/ml)	MIC Range	-	-	-	-	-	-	8->8	-	-	8–32	≤0.125-16
	MIC 50	-	-	-	-	-	-	8	-	-	-	8
	MIC 90	-	-	-	-	-	-	>8	-	-	-	8

## Conclusions

*Trichosporon* is an emerging infection presenting more in invasive forms in recent times which is a matter of concern. Its inherent resistance to commonly used antifungals makes it a more grave infection. Prompt diagnosis and timely management will play a key role in dealing with this fungus.
